# Is integrative therapy of traditional Chinese medicine and progesterone capsule more effective than monotherapies in oligomenorrhea and hypomenorrhea? Evidence based on a multi-center randomized controlled trial and metabolomic profile

**DOI:** 10.1515/jtim-2025-0027

**Published:** 2025-07-08

**Authors:** Xuesong Ding, Tao Tao, Jin Fu, Yu Zhang, Xiaomin Yang, Mulan Ren, Xiaoqin Zeng, Xiaodong Li, Lianglian Tang, Jie Duan, Yan Liu, Jingwen Gan, Yingying Guo, Wanqi Liang, Yu Wang, Wei Xue, Yan Deng, Yanfang Wang, Aijun Sun

**Affiliations:** National Clinical Research Center for Obstetric & Gynecologic Diseases, Department of Obstetrics and Gynecology, Peking Union Medical College Hospital, Chinese Academy of Medical Sciences & Peking Union Medical College, Beijing, China; Department of Reproductive Endocrinology, West China Second University Hospital, Sichuan University; Key Laboratory of Birth Defects and Related Diseases of Women and Children, Ministry of Education, Chengdu, Sichuan Province, China; Gynecology Outpatient Department, The Liuzhou Maternal and Child Health Care Hospital, Liuzhou, Guangxi Zhuang Autonomous Region, China; Department of Obstetrics and Gynecology, Zhongda Hospital Southeast University, Nanjing, Jiangsu Province, China; Department of Gynecology, Guangzhou Women and Children's Medical Center, Guangzhou, Guangdong Province, China; Department of Obstetrics and Gynecology, The First Hospital of Hebei Medical University, Shijiazhuang, Hebei Province, China; Department of Obstetrics and Gynecology, The First Affiliated Hospital of Chongqing Medical University, Chongqing, China; Department of Gynecology, The Maternal and Child Health Hospital of Hubei Province, Tongji Medical College, Huazhong University of Science and Technology, Wuhan, Hubei Province, China

**Keywords:** oligomenorrhea, hypomenorrhea, Chinese herbal medicine, progesterone, combo treatment, metabolomic analysis

## Abstract

**Background and Objectives:**

Oligomenorrhea and hypomenorrhea were common conditions in outpatient visit which affected child-bearing aged women, and the conventional progesterone treatment has been related to several side effect and contradiction, and this study aimed to evaluate the effectiveness and safety of traditional Chinese medicine (TCM), progesterone capsules, and their combination in treating oligomenorrhea and hypomenorrhea *via* both clinical and metabolomic approach.

**Methods:**

A prospective, randomized, multi-center trial has been conducted, and a total of 239 oligomenorrhea and hypomenorrhea were randomly assigned to receive TCM, progesterone capsules (PG), or the combined Chinese and Western medicine (COM) and treated from 3 months, respectively. Scores were recorded at 1^st^ and 3^rd^ month of treatment, while the seral samples for sex hormone and safety parameters were taken before and after management, with adverse events recorded. A post-hoc metabolomic research was further conducted, in which a total of 84 patients' blood sample collected before and after 3-month treatment were screened *via* liquid chromatography-tandem mass spectrometry (LC-MS) and the results were analyzed with machine learning models to identify the differential metabolites, and Kyoto Encyclopedia of Genes and Genomes (KEGG) pathway database and Interactive pathways explore (iPath) database were applied to unveil the potential pathway involved in the treatment.

**Results:**

A total of 220 participants completed the trial treatment, with dropout rate of 11.6%. The Traditional Chinese Medicine Syndrome Scale (TCMSS) scores of each group were significantly lower than those before treatment after 1 month of treatment and after 3 months of treatment. After 3 months of treatment, the COM group had the highest total effective rate (61.54%). The pictorial blood loss assessment chart (PBAC) scores in the TCM group were significantly higher than those before treatment after 1 month of treatment and 3 months after treatment, whereas no difference before and after treatment was observed within PG group. In COM group, the score after 3 months of treatment was significantly higher than that before treatment. In the post-hoc study with metabolomic approach with KEGG pathway enrichment and topological analyses, glycerophospholipid (GPL) metabolism were found significantly altered in all 3 groups. while TCM could potentially improve the clinical symptom *via* tryptophan metabolism.

**Conclusion:**

From the perspective of TCMSS scores, the effect of COM group is more significant. In terms of PBAC scores, sex hormone level, endometrium and the improvement of ovarian function in patients with low ovarian function, the TCM group had a better response. In a comprehensive evaluation, the regimen of TCM alone or COM can significantly improve menstrual flow and treat oligomenorrhea and hypomenorrhea patients. With KEGG pathway enrichment and topological analyses, GPL metabolism were found significantly altered in all 3 groups, and TCM could potentially improve the clinical symptom *via* tryptophan metabolism. Whereas unique pathways influenced under combo treatment suggested a synergetic effect of combination TCM with western medicine in management of oligomenorrhea and hypomenorrhea.

## Introduction

Menstrual disorder is one of the most common clinical symptoms in women, accounting for one-third of all gynecological outpatient visits. Since puberty, about 70% of women have experienced menstrual disorders, which includes shortened or prolonged menstruation and abnormal menstrual flow.^[1]^ Menstrual disorders can be caused by a variety of factors, including stress, nutritional deficiencies, chronic diseases, poor lifestyle, and medical factors.^[2]^ Oligomenorrhea was defined as extended menstrual cycle over 35 days, while hypomenorrhea refers to a condition of decreased or short menstrual periods with blood loss less than 30 mL per cycle, reflecting the malfunction of ovulation.^[3]^ Additionally, the irregular bleeding in women of childbearing age may also indicate a decrease in ovarian reserve function.^[4]^ The repetitive stimulation of endometrium by estrogen in anovulatory cycles may lead to increased risk of hyperplasia even malignancy over long term, as the failure of scanty ovulation to produce enough progesterone to convert the endometrium on a regular basis.^[5]^ Therefore, management of scanty menstruation and decreased menstrual flow not only improves the quality of life and psychological well-being of the patient, but also protects the uterus and fertility.^[6]^

In western medicine, menstrual disorders may be related to irradiation, chemotherapy, autoimmune disorders, and psychological and environmental factors.^[7]^ Hormone therapy can effectively regulate the menstrual cycle and improve menstrual flow, but it requires a thorough history of the patient to uncover the potential contraindications to hormone therapy.^[8]^ In addition, the medicine containing sex hormone can lead to various adverse effects, dizziness and nausea for instance, and the conditions may recur after stopping the drugs.^[9]^ Research has proved that traditional Chinese medicine (TCM) can improve the overall condition of patients’ deficiencies by benefiting qi, tonifying blood, tonifying the kidney and strengthening the spleen, which was suggested to have better long-term efficacy and fewer side effects than hormone therapy.^[10]^ For example, the combination of the formula of tonifying the kidney and activating blood to promote ovulation with Guizhi Fuling capsule and Guishao Tiaojing capsule, with the 82.8%–94.8% of clinical effective rate of improving menstrual disorders.^[6,11,12]^

The TCM formula we interested in is a pure Chinese medicine preparation suitable for symptoms such as dizziness, pale face, and fatigue caused by insufficient qi and blood, and it could replenish blood and Shen essence, and coordinate the functions of viscera.^[13]^ However, no clinical study has evaluated its effects on menstrual disorders. Theoretically, the combination of Chinese and Western medicine (progesterone) can not only quickly regulate menstruation with fewer side effects but also prevent the recurrence of symptoms attributed to the radical treatment at the pathogenesis of the diseases. Therefore, we conducted a comprehensive study to explore the effects of the combination of the TCM and Progesterone capsules (PG) in oligomenorrhea and hypomenorrhea compared to monotherapy *via* randomized controlled trial.

In addition, more and more studies have adopted metabolomics to explain the overall mechanism of action of TCM.^[14]^ Metabolomics is a research method developed after genomics and proteomics, which can reveal the exact metabolic changes of the organism in a certain disease state. It is possible to clarify the biomarkers of the organism in a specific state, which may provide a new direction for clinical diagnosis and treatment.^[15]^ Therefore, a post-hoc analyses were further conducted to explore the underlying mechanism of these regimens *via* non-targeted liquid chromatography-tandem mass spectrometry (LC-MS).

## Methods

### Participants

Based on the preceding clinical trial, all subjects were obtained from the outpatient clinics for menstrual disorders of Peking Union Medical College Hospital, Guangzhou Women’s and Children’s Medical Center, the First Hospital of Hebei Medical University, Hubei Provincial Maternity and Child Health Hospital, the Second Hospital of West China of Sichuan University, Liuzhou Maternity and Child Health Hospital, and the Zhongda Hospital of Southeast University from June 2022 to December 2022. This multi-center prospective randomized controlled trial was registered at ClinicalTrials. gov (http://www.clinicaltrials.gov; Registration No. NCT05312190). The trial was approved by the Ethics Committee of Peking Union Medical College Hospital (approval No. HS-3365D).

Women with menstrual disorders who are clinically diagnosed as having oligomenorrhea or hypomenorrhea, which refers to a prolonged menstrual cycle, > 35 days or menstrual flow of less than 30 mL, respectively. Inclusion criteria were (1) patients aged between 25 and 40 years; (2) patients meeting the modern diagnostic criterion of oligomenorrhea and hypomenorrhea; and (3) patients who provided informed consent and volunteered to participate. Exclusion criteria included (1) patients with ovarian dysfunction due to ovarian surgery or chemoradiotherapy; (2) patients with cervical and or uterine adhesions; (3) patients with endometriosis, uterine fibroids, and amenorrhea galactorrhea syndrome; (4) patients taking hormones or immunosuppressive drugs; (5) patients with severe or unstable heart, liver, kidney, endocrine, blood and the other physical diseases; (6) patients who were contradictory to take prescribed medicine, *e.g*. those with breast cancer or other sex hormone-dependent malignant tumors, or a history of thromboembolic diseases; (7) patients with allergies or severe adverse reactions to the drugs used in the trial; (8) patients with a history of drug use to regulate menstruation in the past 3 months; and (9) patients with substance abuse or dependence (alcohol, tobacco or drugs) in the past 3 months.

### Sample size

We performed a t-sample t-test power analysis *via* PASS software (version: 15.0.13 NCSS LLC, Kaysville, UT, USA), the analysis showed that with a sample population of 198 women, the analysis would be able to detect a 2-point reduction weighed by score (α = 0.05 and 1 - β = 0.90). a total of 234 patients with oligomenorrhea and hypomenorrhea were required with a dropout rate of 15% (78 in each group).

### Intervention and follow-up

The intervention assignment information for each randomization number was sealed in separate opaque envelopes by an investigator, and medication was administered accordingly. Participants were randomly allocated into 3 group, namely TCM group, PG group and the combination of TCM and PG (COM) group. (1) TCM group: Chinese patent medicine with main ingredients: Sheep Placenta, Pearl, Red Ginseng, Astragalus, Wolfberry, Angelica Dahurica, Coix Seed, Ginger, Jujube. 10 mL bid orally; (2) PG group: progesterone capsule 200 mg qd* 10 days orally starting from the second half of the menstrual cycle; (3) COM group: combined therapy of TCM and progesterone simultaneously.

Medication was given for 3 months based on clinical experiences and Traditional Chinese Medicine Syndrome Scale (TCMSS) scores, pictorial blood loss assessment chart (PBAC) scores, 36-item Short Form Health Survey (SF-36) scores were recorded at 1^st^ and 3^rd^ month of treatment, while the seral samples for sex hormone and safety parameters and ultrasonography for endometrial thickness were taken before and after management, with adverse events recorded. In post-hoc study, a total of 84 patients’ blood sample were retrieved (30, 24, 30 for each group, respectively), and were prepared to LC-MS in same bench.

#### Collection of clinical information and serum samples

TCMSS scores were determined according to the Guiding Principles for Clinical Research of New Chinese Medicine and Criteria for the Diagnosis and Efficacy of Diseases in TCM.^[16]^ The menstrual TCM Syndrome contains main and secondary symptoms. A lower score indicated a better assessment of menstrual health. Based on the severity of the symptoms, the main symptoms were assigned 0 (normal menstruation, menstrual blood was bright red), 2 (the amount of menstrual blood loss [MBL] was 20–30 mL, the duration of bleeding lasted for 2–3 days, the normal menstrual cycle continued < 3 months, and menstrual blood was light dull-red), 4 (MBL < 20 mL, duration of 1–2 days, menstrual cycle prolonged to 35 days–2 months, and menstrual blood was thin and dull-red) and 6 (spotting, duration of < 1 day, menstrual cycle prolonged to 3–5 months, and menstrual blood was water-like and dull-red).

Secondary symptoms were assigned 0 (asymptomatic), 1 (occasional symptoms), 2 (frequent symptoms but normal life), and 3 points (frequent symptoms and affected life). Patients were required to compare the descriptions of the main and secondary symptoms on the scale and record the total menstrual TCMSS scores at the end of menstruation.

Total Effective Rate of TCM was defined as: (1) recovery: curative effect index 95%; (2) markedly effective: curative effect index between 95%–70%; (3) effective: curative effect index between 70% and 30%; and (4) ineffective: curative effect index < 30%. Curative effect index = (TCMSS scores before treatment-TCMSS scores after treatment) /TCMSS scores before treatment×100%]. The total effective rate = ([recovery cases + obvious effective cases + effective cases] /total cases) × 100%.

Patients were required to record the number of sanitary pads used in the PBAC and the degree of menstrual blood infiltration. The light was defined as the blood area, which accounted for 1/3 of the whole sanitary pad. Medium was defined as the blood area accounting for 1/3–3/5 of the entire sanitary pad. Heavy was defined as the entire sanitary pad being blood-stained.^[16]^ Light, medium, and heavy bloodstains were scored as 1, 5, and 20 points, respectively. Each small clot earned 1 point, and each large clot earned 5 points.

SF-36 contains 36 items. The second item was used to evaluate patients’ health changes in the past year, and the other 35 items assessed the patients’ physical and mental health through 8 dimensions: (1) physical functioning; (2) role limitations due to physical problems; (3) bodily pain; (4) general health; (5) vitality; (6) social functioning; (7) role limitations due to emotional problems; and (8) mental health.^[17]^ To score the items in any domain, the scores of the items are summed up and then converted into a scale from 0 (worst) to 100 (best). The higher the score, the better the quality of life.^[18]^

Fasting blood samples were collected from the patients at baseline and 3 months after treatment. The levels of sex hormones were collected during the 2nd and 3rd days of the menstrual cycle from women who showed regular menstruation or on any day for amenorrheic patients. The level of sex hormone including follicle stimulating hormone (FSH), luteinizing hormone (LH), Estradiol (E2), anti-Mullerian hormone (AMH) were measured with chemiluminescence immunoassay using a Beckman Coulter DXI 800 (Beckman Coulter Inc. Brea, CA, USA).

The thickness of the uterine endometrium was monitored by the same trained physician at baseline and 3 months after treatment with an 8.0-mHz vaginal probe (LOGIQ Expert; GE, Milwaukee, USA). Transvaginal ultrasonography was performed twice on the day 1 to 3 after menstruation, measured in the sagittal plane and recorded the thickest point between the two basal layers of the anterior and posterior uterine walls.

### Safety monitoring

1. Record the adverse reactions of the drug;
2. General physical examination items, such as heart rate, blood pressure, weight, *etc*.;
3. Fasting blood samples were collected at baseline and 3 months after treatment. Safety indices, such as complete blood count and hepatic and renal function, were also collected during the 2nd and 3rd days of the menstrual cycle from women who showed regular menstruation or on any day for amenorrheic patients. Blood indices were measured using standard automated procedures with Beckman & Coulter AU automated chemistry analyzers (AU5800, Beckman Coulter Inc. Brea, CA, USA). Reactions or adverse events (including serious reactions) were monitored.

### Sample preparation for LC-MS

Before and after 3-month treatment, 8 mL of blood was collected from the elbow vein in the early morning in limosis, and centrifuged for 15 min at 4 ℃, 1500 ×*g* to collect the plasma, which were frozen at -80 ℃ for storage. The preserved plasma samples were thawed at 4°C, shaken and mixed well, 100 μL of plasma sample was aspirated, 400 μL of methanol was added, vortexed and shaken for 2 min to precipitate the proteins, and then centrifuged (13,000 ×*g* at 4°C for 20 min), and the supernatant of 400 μL was transferred. Reconstituted solvent acetonitrile-water (1: 4 v/v) was added, vortex for 2 min to dissolve, centrifuge (4°C, 13,000 ×*g*, 30 min). The supernatant was collected in a 2-mL vial for analysis. QC samples were generated every 5 prepared sample described above.

### LC-MS analysis

A Waters ACQUITY ultra-high performance liquid chromatography system (Waters Corp, Milford, MA, USA) with an autosampler was used for the analysis, Chromatographic analysis was performed on a Boston Green ODS BEH-C18 column (100×2.1 mm, i.d. 1.7 μm, Waters Corp, Milford, MA, USA). The column temperature was 40 ℃ and the sample chamber temperature was 4 ℃. The flow rate was 0.4 mL/min and the injection volume was 10 μL. The mobile phases of chromatography were composed of two phases, 0.1% (v/v) formic acid-water in phase A and 0.1% (v/v) formic acid-acetonitrile in phase B.

For mass spectrometry analysis: ESI-Q-TOF-MS was acquired in negative ion mode; capillary voltage 3000 V, cone bore voltage 30 V, dissolvent gas temperature 350 ℃, ion source temperature 110 ℃, dissolvent gas flow rate of 600 L/h, cone bore gas flow rate of 50 L/h; acquisition time 0–23 min, scanning range m/z100–1500, 0.1 s per scan, two scanning intervals of 0.02 s; the detection process using leucine enkephalin (200 μg/L) as a tuning solution for real-time correction.

### Statistical analyses

All statistical analyses for clinical parameters were performed using SPSS software version 25.0 (IBM Inc., USA) and Excel. TCMSS scores, SF-36 scores, sex hormones, thickness of endometrium and safety indices are normally distributed, expressed as mean ± standard deviation and examined using analysis of variance and Bonferroni correction for multiple comparisons among groups. PBAC scores are skewedly distributed results, expressed as medians (interquartile range) and differences among groups analyzed by Kruskal–Wallis. Comparisons within the same group before and after 3-month treatment were performed using a paired t-test for normally distributed data and Wilcoxon signed-rank test for skewed data, respectively. The total effective rate of TCM syndrome was analyzed using the Chi-square test. Statistical significance was set at *P* < 0.05.

After plasma samples were separated by chromatography and collected by mass spectrometry, the raw mass spectrometry data files were generated, and a three-dimensional data matrix containing retention time, mass-to-charge ratio, and peak area was generated by filtering noise peak detection and peak matching using the Masslynx (Waters, USA) software package, which was used for multivariate statistical analysis and focusing analysis of the characteristic indexes at a later stage. Multivariate statistical analysis was performed using MetaboAnalyst 6.0 (www.metaboanalyst.ca/MetaboAnalyst/home.xhtml). Partial least squares discriminant analysis (PLS-DA) and orthogonal partial least squares cluster analysis (OPLS-DA) were used to cluster the data. The list of metabolites showing retention time, mass-to-charge ratio, and response strength was obtained by selecting the variables that contributed more to the variance based on the variable important in projection (VIP) obtained from the OPLS-DA model and the paired samples t-test. The differential metabolites were imported into HMDB (http://www.hmdb.ca/), KEGG online database (www.genome.jp/kegg/), and Interactive pathways explore 3.0 (iPath 3.0, http://pathways.embl.de) to search and identify potential biomarkers and related mechanisms with biological significance.

## Results

### Study subjects and baseline characteristics

**Figure 1:**
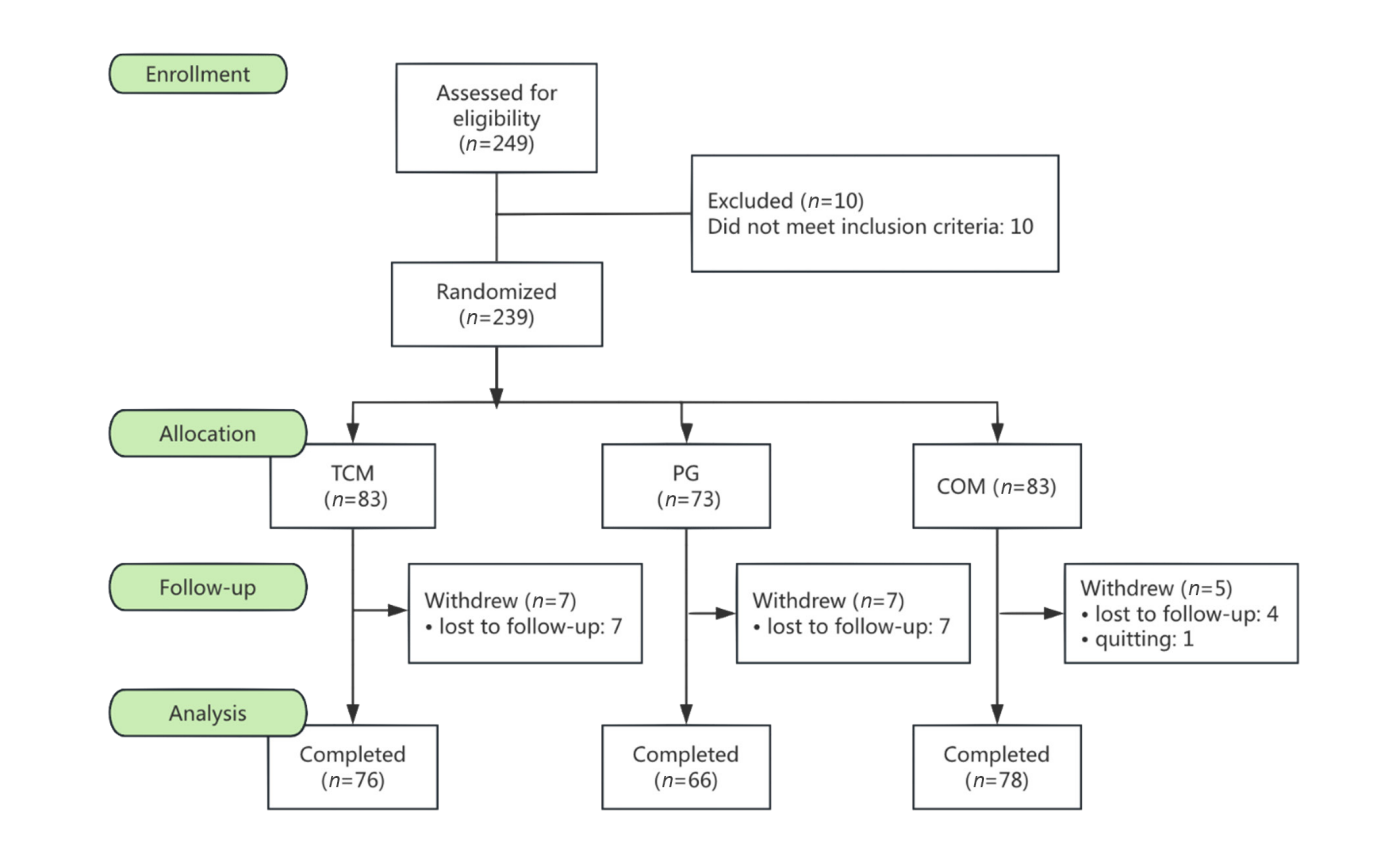
The process of study from screening to completion during the 12 weeks.

According to the inclusion criteria, 239 out of 249 met the initial criteria. The study subjects were randomly separated into the TCM group, PG group and COM group. Eventually, there are 220 subjects who completed the trial treatment, so the shedding rate was 11.6%. Besides, 19 subjects early withdrew from the trial: 7 patients in the TCM group, 7 patients in the PG group and 5 patients in the COM group required withdrawal without reason early (Figure 1).

The baseline characteristics of all randomized patients are presented in Table 1. There were no significant differences between groups except AMH.

**Table 1:** Baseline characteristics of the study participants among the three groups (Mean ± SD)

group	N	Age (year)	BMI (kg/m^2^)	FSH (IU/L)	LH (IU/L)	E2 (pg/L)	AMH (μg/L)	Inner membrane thickness (cm)
TCM	76	32.79±4.85	20.27±4.98	7.74±3.69	5.04±3.33	49.71±43.43	3.73±3.34	0.58±0.20
PG	66	31.02±5.01	20.26±6.18	7.38±4.3	6.87±6.09	68.55±78.13	5.18±3.99	0.64±0.35
COM	78	31.53±4.72	20.59±5.88	7.00±3.59	7.29±6.00	48.38±33.95	5.97±5.14^*^	0.59±0.22

FSH: follicle stimulating hormone; LH: luteinizing hormone; E2: estradiol; AMH: Anti-Mullerian hormone; BMI: body mass index; TCM: traditional Chinese medicine; PG: progesterone; COM: the combined Chinese and Western medicine.

### TCMSS scores

The paired comparison results showed that the TCMSS scores of 1 month and 3 months decreased respectively compared to those of pre-test in three group. (TCM group: *P*1 < 0.01, *P*3 < 0.01; PG group: *P*1 < 0.01, *P*3 < 0.01; COM group: *P*1 < 0.01, *P*3 < 0.01), but only in the TCM and COM group the scores of 1 month and 3 months decreased successively (TCM group: *P* < 0.01; COM group: *P* < 0.01).

On the other hand, the changes in TCMSS scores in different periods are analyzed by repeated measures ANOVA are presented in Table 2. The outcomes reflects that the scores of the COM group at 3 month is lower compared to those in the PG group (*P* < 0.05).

**Table 2:** The comparison of TCMSS scores

groups	Follow-up time	*N*	TCMSS scores	The reduction of TCMSS scores	The rate of reduction
	Pre-treatment	76	11.68±5.37	——	——
TCM	After 1 month of treatment	76	9.26±4.85^**^	2.42±4.98	0.2 (0,0.33)
	After 3 months of treatment	76	6.87±3.96^**##^	4.82±5.34	0.4 (0.10,0.59)
	Pre-treatment	66	11.38±4.86	——	——
PG	After 1 month of treatment	66	8.67±4.38^**^	2.71±3.97	0.19 (0,0.41)
	After 3 months of treatment	66	8.08±4.31^**^	3.3±4.39	0.36 (0.05,0.52)
	Pre-treatment	78	11.56±4.31	——	——
COM	After 1 month of treatment	78	8.94±4.64^**^	2.63±3.98	0.2 (0,0.45)
	After 3 months of treatment	78	6.4±4.35^**##▲^	5.17±5.34^★^	0.41 (0.2,0.73)

^**^P<0.01 *vs*. Pre-treatment scores in the same group; ^##^*P*<0.01 *vs*. Score after 1 month of treatment in the same group; ^▲^*P*<0.05 *vs*. PG group after 3 months of treatment. ^★^*P*<0.05 *vs*. reduction of score in PG group after 3 months of treatment. TCM: traditional Chinese medicine; PG: progesterone; COM: the combined Chinese and Western medicine.

When we calculated the reduction of scores at each follow-up, we observed that COM group had significantly higher reduction than those in the Western medicine. (*P* < 0.05).

### The comparison of the clinical efficacy of TCMSS scores

However, when we analyzed the clinical efficacy at each follow-up (the reduction of TCMSS scores over 30% reckoned effective), we observed that after 3 months of treatment, the clinical efficacy of all the groups was respectively higher than those of 1 month after treatment (Table 3). Although the efficacy of the combined group is always the highest among the therapies (1 month: 42.31%, 3 months: 61.54%), no distinct differences were found in all these therapies (Table 3).

**Table 3:** Clinical efficacy of three groups at different follow-up

Groups	Time	Recovery (%)	Markedly effective (%)	Effective (%)	Total effective (%)	Ineffective (%)	Pearson X^2^	*P*
TCM	After 1 month of treatment	1(1.32)	3(3.95)	19(25.00)	23(30.27)	53(69.74)	11.73529	0.0006
	After 3 months of treatment	1(1.32)	9(11.84)	35(46.05)	45(59.21)	31(40.79)		
PG	After 1 month of treatment	2(3.03)	3(4.55)	16(24.24)	21(31.82)	45(68.18)	5.241541	0.0217
	After 3 months of treatment	2(3.03)	4(6.06)	29(43.94)	35(53.03)	31(46.97)		
COM	After 1 month of treatment	1(1.28)	5(6.41)	27(34.62)	33(42.31)	45(57.69)	5.033086	0.0246
	After 3 months of treatment	5(6.41)	17(21.79)	26(33.33)	48(61.54)	30(38.46)		

TCM: traditional chinese medicine; PG: progesterone; COM: the combined Chinese and Western medicine.

### PBAC scores

At 1 and 3 months, the PBAC scores of the TCM group increased significantly (*P*1 < 0.05, *P*3 < 0.01) and the scores of 3 months are distinctly higher than 1 month. (*P* < 0.01). As for the Combined group, PBAC scores showed a statistically significant improvement until 3 months long. However, the PBAC scores of PG was no significant change during the treatment period. Comparing their improvement trend among groups, significant differences were observed between PG and TCM group (Table 4).

**Table 4:** PBAC scores of three groups at each follow-up

Groups	Time	*N*	PBAC scores	The reduction of TCMSS scores	The rate of change
TCM	Pre-treatment	76	23.00(12.00, 46.75)	——	——
	After 1 month of treatment	76	28.00(17.00, 55.75)^*^	5(-4, 13.5)	0.24(-0.13, 0.98)
	After 3 months of treatment	76	40.50(19.00, 78.50)^**##^	13(-2, 31)^▲^	-0.54(-1.47, 0.08)^▲^
PG	Pre-treatment	66	43.50(17.75, 68.25)	——	——
	After 1 month of treatment	66	50.50(22.75, 81.75)	3(-5.75, 17.75)	3(-5.75, 17.75)
	After 3 months of treatment	66	44.50(20.00, 82.25)	0.5(-12.75, 21.5, )	0.5(-12.75, 21.5, )
COM	Pre-treatment	78	31.50(16.25, 71.00)	——	——
	After 1 month of treatment	78	35.50(21.50, 73.25)	4.5(-13.25, 19)	0.24(-0.24, 1.16)
	After 3 months of treatment	78	47.00(25.50,82.25)^*^	10(-18, 33)	0.48(-0.29, 1.71)

^*^P<0.05, ^**^P<0.01 *vs*. Pre-treatment scores in the same group; ^##^*P*<0.01 *vs*. Score after 1 month of treatment in the same group; ^▲^*P*<0.05 *vs*. PG group after 3 months of treatment. TCM: traditional chinese medicine; PG: progesterone; COM: the combined Chinese and Western medicine.

### Other secondary endpoints

Only the SF-36 score of the PG group was significantly higher compared with baseline scores (*P* < 0.05). However, there were no significant differences among groups at each follow-up (Table 5).

**Table 5:** SF-36 scores of three groups

Groups	Time	*N*	SF-36 scores	The reduction of scores	The rate of change
TCM	Pre-treatment	76	115.37±13.03		
	After 3 months of treatment	76	116.53±10.33	-1(-5, 6)	-0.008 (-0.046, 0.0467)
PG	Pre-treatment	66	112.68±13.97		
	After 3 months of treatment	66	116.86±10.86^*^	-3(-12.5, 2)	-0.026 (-0.123, 0.017)
COM	Pre-treatment	78	118.24±12.51		
	After 3 months of treatment	78	118.48±11.45	-1.5(-5.66, 5)	-0.012 (-0.049, 0.041)

^*^P < 0.05 *vs*. PG group after 3 months of treatment. TCM: traditional Chinese medicine; PG: progesterone; COM: the combined Chinese and Western medicine.

### Changes in sex hormone levels and endometrium thickness

After 3 months of treatment, FSH, E2, LH were significantly increased in TCM group (*P* < 0.05) but not in the other two groups. And the rise of PRL level was observed in PG after 3 months (*P* < 0.05). In addition, the androgen level increased significantly after 3 months of treatment (*P* < 0.05) in combined group. However, no distinct between-treatment differences were found in sex hormone level change.

As with the endometrium thickness, only the endometrium thickness of TCM group after 3 months is significantly higher than baseline, but no differences among groups were found at each follow-up (Table 6).

**Table 6:** Changes in sex hormone levels and endometrial thickness in the three groups

Groups	*N*	Time	Sex hormone						Endometrial thickness (cm)
			FSH (IU/L)	LH (IU/L)	E2 (pg/mL)	AMH (μg/L)	T (ng/mL)	PRL (ng/mL)	
	76	Pre-treatment	7.74±3.69	5.04±3.33	49.71±43.43	3.34±9.64	3.73±3.34	13.84±7.66	0.58±0.20
TCM	76	After 3 months of treatment	7.57±7.83^*^	6.79±6.71^*^	59.91±43.05^*^	2.77±7.85	3.74±3.19	13.61±7.97	0.64±0.26^*^
	66	Pre-treatment	7.38±4.30	6.87±6.09	68.55±78.13	7.20±17.12	5.18±3.99	15.25±14.02	0.64±0.35
PG	66	After 3 months of treatment	6.63±2.70	6.59±5.65	59.23±55.82	5.29±11.91	5.14±4.17	15.81±12.48^*^	0.61±0.29
	78	Pre-treatment	7.00±3.59	7.29±6.00	48.38±33.95	5.60±12.58	5.97±5.14	12.79±6.49	0.59±0.22
COM	78	After 3 months of treatment	7.73±7.07	6.79±4.77	51.77±45.31	4.33±10.05	5.71±4.53^*^	13.25±7.65	0.56±0.20

^*^P<0.05 *vs*. Pre-treatment scores in the same group; TCM: traditional Chinese medicine; PG: progesterone; COM: the combined Chinese and Western medicine.

### Changes in sex hormone levels in patients with ovarian insufficiency

We selected patients whose FSH > 10 IU / L or AMH < 1.11 μg / L for further analysis and discovered that the FSH level significantly decrease in both TCM group and PG group after 3 months of treatment. Due to the limited case of combined group, no significant change of FSH level were observed in this group.

Comparing with baseline, AMH level increased significantly only in the TCM group (*P* < 0.05), and the AMH level of TCM group is much higher than PG group after 3 months of treatment (Table 7).

**Table 7:** Improvement of FSH and AMH levels in patients with ovarian insufficiency

Groups	Time	*N*	FSH (IU/L)	Example number	AMH (μg/L)
TCM	Pre-treatment	12	14.59±3.46	15	0.62±0.34
	After 3 months of treatment		9.33±3.62^**^		1.00±0.72^*▲^
PG	Pre-treatment	12	13.43±6.70	10	0.39±0.32
	After 3 months of treatment		7.03±3.01^**^		0.38±0.31
COM	Pre-treatment	3	11.90±1.70	8	0.47±0.24
	After 3 months of treatment		12.97±5.33		0.7±0.63

^*^P<0.05, ^**^P<0.01 *vs*. Pre-treatment scores in the same group; ^▲^*P*<0.05 *vs*. PG group after 3 months of treatment. TCM: traditional chinese medicine; PG: progesterone; COM: the combined Chinese and Western medicine.

### Safety indices and adverse events

In all the groups, safety indices did not significantly change after 3 months of treatment. And no between-treatment difference differences were found at each follow-up time point (Table 8).

**Table 8:** Safety indices of the three groups

Groups	*N*	Time	ALT (U/L)	TBiL (μmol/L)	DBiL (μmol/L)	Cr (μmol/L)	Urea nitrogen (mmol/L)	UA (μmol/L)
	76	Pre-treatment	16.17±12.23	11.3±4.86	3.93±2.00	58.89±7.59	4.25±1.11	294.20±77.98
TCM	76	After 3 months of treatment	13.97±6.13	11.35±5.91	3.92±2.06	57.72±12.08	4.86±5.21	290.40±64.23
	66	Pre-treatment	16.30±13.30	10.71±4.81	3.68±2.16	58.56±10.59	4.28±1.04	304.89±68.36
PG	66	After 3 months of treatment	15.95±11.33	10.22±3.68	3.53±1.43	58.00±9.59	5.11±6.98	306.92±80.42
	78	Pre-treatment	17.56±18.15	11.58±9.59	4.09±5.55	56.77±8.44	4.18±1.08	297.29±73.19
COM	78	After 3 months of treatment	15.97±14.95	11.16±5.31	3.69±1.74	55.72±10.06	5.01±7.34	295.18±68.29

TCM: traditional Chinese medicine; PG: progesterone; COM: the combined Chinese and Western medicine.

During the treatment, the patients in the TCM group had no medication-related adverse reactions. Two patients in the PG group were affected by headache and dizziness, chest discomfort and nausea. And one in the COM group had heavy menstrual bleeding during medication and exited the trial. There was no significant difference between groups in the incidence of adverse reactions (*P* > 0.05) (Table 9).

**Table 9:** Comparison of the incidence of adverse reaction among the three groups

Groups	No reaction adverse	Adverse reaction	X^2^	*P*
TCM	76	0		
	100.00%	0.00%		
PG	64	2	2.418	0.299
	97.00%	3.00%		
COM	77	1		
	48(61.54%)	30(38.46%)		

TCM: traditional Chinese medicine; PG: progesterone; COM: the combined Chinese and Western medicine.

### Serum metabolic profiles by LC-MS

**Figure 2:**
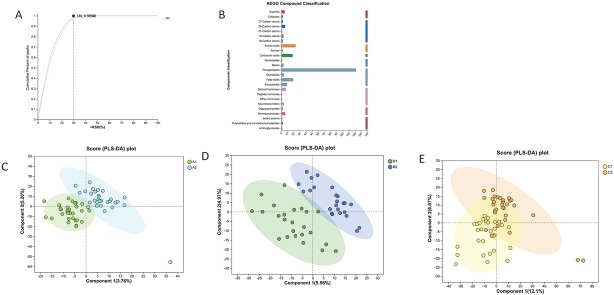
(A) the relative standard deviation distribution plot (B) Identified compounds classification with Kyoto Encyclopedia of Genes and Genomes (KEGG) (C) PLS-DA analysis in pre- and post-treatment group of TCM (D) PLS-DA analysis in pre- and post-treatment group of PG (E) PLS-DA analysis in pre- and post-treatment group of COM.

In post-hoc analysis, a total of 84 patients’ serum sample were collected for the post-hoc analysis (30 in TCM group; 24 in PG group, and 30 in COM group), and blood samples were collected before or after 3-month management. A liquid chromatography coupled to a tandem mass spectrometry system was chosen to perform the non-targeted ^[19]^ profile of serum samples, and the relative standard deviation (RSD) distribution of raw data in quality control samples was plotted in Figure 2A. With data pre-treatment, the peaks of cumulative percent reach over 99% at 30% of RSD, suggesting a robust method and constant quality of LC-MS measurement. A total of 5478 and 7666 peaks were detected in this bench, from which 1016 and 1394 metabolites were identified in positive ion mode and negative ion mode respectively. In addition, the majority of identified compounds within KEGG databases were lipid, including 121 phospholipids, 19 fatty acids, 9 eicosanoids, and 4 glycolipids (Figure 2B).

The supervised PLS-DA models were trained to identify the pattern between pre-treatment and post-treatment of within 3 groups, *i.e*. TCM, PG, and COM group (Figure 2C–E). The overall trend of distribution among different groups was delineated *via* PLS-DA, and the distinct separation before and after 3-month treatment can be observed within each group, especially for TCM group and PG group. Premutation tests were performed and plotted to evaluate these models. All the intercept of each Q2 were negative, and R2Y of each model was 0.805, 0.825 and 0.568, respectively, reflecting a relatively reliable and accurate predictive performance. The obvious separation pattern between before and after treatment within 3 different groups indicated drastic alternations of seral metabolic profile introduced by treatment which may related to the improvement of clinical symptom in patients with oligomenorrhea or hypomenorrhea.

### Identification of differential metabolites

**Figure 3:**
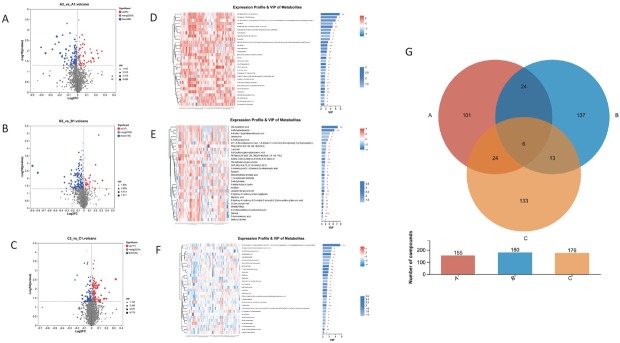
Volcano plots of differential metabolites in TCM group (A), PG group (B) and the COM group (C), red dots indicate up-regulated metabolites with *P* < 0.05; blue dots indicate down-regulated metabolites with *P* < 0.05; VIP value is indicated as diameter of dots, with numeric illustration at legends. Heatmaps of the top 30 differential metabolites in TCM group (D), PG group (E) and the COM group (F), the color indicates the relative expression level of the metabolites in this group of samples, with bar color indicates the significance of the difference before and after treatment, and the smaller the *P* value is, the darker the color is. ^*^P < 0.05, ^**^P < 0.01, ^***^P < 0.001. (G) Venn plots of differential metabolites from TCM group, PG group, and the COM group, with numeric label of common and unique differential metabolites among three groups.

The expression pattern of metabolites detected in LC-MS were screened for potential targets through TCM or progesterone or the combo management, according to the VIP score > 1 and *P* value < 0.05. A total of 155, 180 and 176 differential metabolites were identified in TCM group, PG group and COM group, respectively (Figure 3G). 6 metabolites shared importance in all three regimens, including L-Isoleucine, 5-Taurinomethyl-2-thiouridine, Brinzolamide, and 2-Cyanoethyl dihydrogen phosphate. Additionally, 30 metabolites involved in both TCM and progesterone monotherapy, whereas 133 more differential metabolites were identified exclusively for combo treatment.

In TCM group, 99 metabolites were down-regulated after 3-month treatment whereas 56 metabolites were up-regulated (Figure 3A). Leurubicin was found as one of the top 4 altered metabolites in TCM treatment. Leurubicin, a potential target of antitumor agency, is an N-L-leucyl prodrug of the anthracycline doxorubicin, with antineoplastic activity. Leurubicin is converted to its active form doxorubicin in or on tumor cells by hydrolytic enzymes. Meanwhile, dibutylphthalic acid and 3-Methyldioxyindole were identified as top 2 differential compound in progesterone treatment. Dibutyl phthalate is a suspected endocrine disruptor, and it is a commonly used plasticizer and also used as an ectoparasiticide. [10, 13-Dimethyl-17-(1-sulfooxyethyl)-2, 3, 4, 5, 6, 7, 8, 9, 11, 12, 14, 15, 16, 17-tetradecahydro-1H-cyclopenta [a] phenanthren-3-yl] hydrogen sulfate, as one the of top differential metabolites in combo treatment, belongs to the class of organic compounds known as sulfated steroids.

Taken together, 37 and 143 of metabolites were significantly up- and down- regulated in progesterone treatment. Comparing with TCM group, more differential metabolites were decreased in post-treatment group in PG group, and fold change of two metabolites, dibutylphthalic acid and 3-methyldioxyindole, were over 1.5 (Figure 3B). In addition, 111 of differential metabolites were increased after combined treatment of TCM and progesterone, meanwhile 65 of them were lower (Figure 3C). The expression profile of top 30 differential metabolites from three groups with highest rank in VIP score were plotted with heatmaps in Figure 3 D–F, and a discrete pattern can be observed between pre-treatment and post-treatment individual samples.

### Pathway enrichment analyses

**Figure 4:**
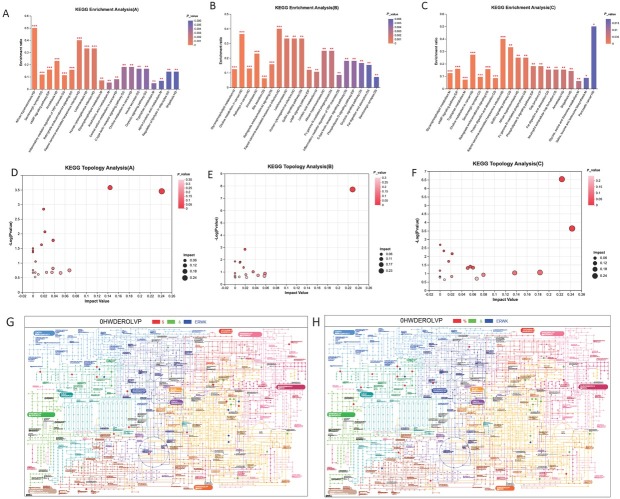
KEGG enrichment analysis of the differentially metabolites in TCM group (A), PG group (B) and the COM group (C). names of involved pathway are labelled at x axis, and enrichment ratio are indicated as the height of bar, ^*^P < 0.05, ^**^P < 0.01, ^***^P < 0.001. KEGG topology analysis of the differentially metabolites in TCM group (D), PG group (E) and the COM group (F). Each bubble in the figure represents a KEGG pathway; the horizontal axis represents the size of the relative importance of the metabolite in the pathway Impact Value; the vertical axis represents the enriched significance of the metabolite’s involvement in the pathway-log10 (P value); the size of the bubbles represents the Impact Value; the bigger the bubbles, the greater the importance of the pathway. the iPath (interactive Pathways Explorer) pathway comparisons between TCM group and COM group (G); between PG group and COM group (H), blue nodes represent the pathways annotated for the two metabolic sets together.

With differential metabolites determined by VIP score > 1 and *P* < 0.05, the KEGG online database was applied to pathway enrichment analysis to explores the most relevant pathway underpinning symptom improvement. Enrichment ratio refers to the relative magnitudes of differential metabolites detected aforementioned and background number in KEGG database, and the enrichment analysis screens the potential pathway based on enrichment ratio and adjusted p values. The involved pathways in TCM group, PG group and the COM groups were illustrated in Figure 4 A–C, in rank of p value from low to high. Additionally, based on relative-betweenness centrality method, the topology analyses were performed (Figure4 D–F, TCM, PG, COM, respectively). In plots D–F, the dimeter of bubble was positively corelated to pathway impact value. Results suggested glycerophospholipid (GPL) metabolism, essential in lipid metabolism and cell Signal transduction, were significantly altered in all 3 groups. while TCM could potentially target tryptophan metabolism; and in combined with progesterone.

Two additional pathways were uniquely influenced under combo treatment, namely Riboflavin metabolism categorized in metabolism of cofactors and vitamins and caffeine metabolism categorized in biosynthesis of other secondary metabolites. It was reported that regulatory effects of riboflavin on lipid metabolism included inhibiting cholesterol biosynthesis, maintaining normal hepatic transport of lipids, lowering triglyceride, free fatty acid, low-density lipoprotein (LDL) levels, and preventing lipid peroxidation and lipid accumulation, suggesting a role for riboflavin as a primary or adjunctive drug in the treatment of lipid metabolism-related diseases such as cardiovascular diseases. It has been identified SLC22A14 is a riboflavin transporter protein located in the inner membrane of mitochondria in the middle section of spermatozoa. Deficiency of this protein interferes with flavinase function, thereby inhibiting oxidative phosphorylation and reorganizing sperm energy metabolism, leading to male sterility.^[20]^

iPath 3.0 was also implied to uncover the underlying metabolite pathway involved in treatments. The differential metabolites derived from TCM group and PG group in comparison with that from the COM group were analyzed in separately, and the map was illustrated in Figure 4G and H respectively. the common metabolite pathways involved in both TCM and COM group were amino acid metabolism, lipid metabolism. Whereas that in comparison between western and combo treatment, less common pathways were found which distributed largely in amino acid metabolism. Intriguingly, the alternation in certain processes of amino acid metabolism were found exclusive in the COM group, suggesting a synergetic effect of the combined therapy.

## Discussion

Oligomenorrhea or hypomenorrhea were two most common conditions affecting child-bearing aged women, and the reduced menstrual flows could result in abnormal uterine bleeding during uncomplete or desynchronized shedding of endometrium and patients’ mental burden of early ovarian insufficiency. PG, a natural steroid hormone, was one of the conventional regimens in PG to management decreased flow or irregular menstrual cycle.^[21]^ However, the major adverse effects of hormonal therapy were breakthrough bleeding, dizziness and discomfort of breasts, which may lead to self-discontinuation of medicine and worsening of irregular bleeding. Meanwhile, the TCM has long been prescribed to benefiting Qi and nourishing blood, tonifying the kidney and strengthening the spleen, which is the indication for oligomenorrhea and hypomenorrhea in TCM theory, and with minimal contradiction and side effect.

The TCM regimen applied in this research is a Chinese medicinal preparation for dizziness, pallor, fatigue, lack of breath and laziness due to insufficient qi and blood, and its main ingredients are sheep placenta, pearl, red ginseng, wolfberry, Astragalus, Angelica dahuricus, ginger and jujube.^[22]^ The Chinese wolfberry (Lycium barbarum) has the effects of nourishing the liver and tonifying the kidneys, replenishing essence, calming the mind and nourishing the blood, while ginseng and astragalus have the effects of enhancing immunity, antioxidant, alleviating neuronal apoptosis and metabolic failure.^[23,24]^ Pharmacological studies have shown that it can promote hematopoietic function and increase local perfusion, and is effective in the treatment of menstrual disorders caused by psychosomatic factors or ovarian insufficiency. Several studies have confirmed that TCM regimen has a certain degree of efficacy and a high degree of safety in the treatment of patients with anemia.^[25]^

### Pharmacological action

The main components of the TCM regime are sheep placenta, pearl, red ginseng, barbary wolfberry seed, Astragalus root, angelica dahuricae, coix seed, ginger, jujube. Among them, sheep placenta contains a variety of high efficiency biological active factors, can benefit kidney qi, tonic blood thus promotes angiogenesis blood circulation.^[26]^

Ninety-one of pearl matrix proteins could be classified into seven categories by their potential medical functions including wound healing, osteogenic property, antioxidant activity, neuro-regulation effects, skin lightening effect, anti-inflammatory and anti-apoptotic effects and other immunomodulatory property.^[27]^

Ginsenoside Rg 1, the main active ingredient of red ginseng, is a kind of phytoestrogen that can exert estrogen-like activity on human breast cancer cells (MCF 7) without binding the estrogen receptor.

Barbary wolfberry Son, which mainly including Barbary wolfberry polysaccharide (U), flavonoids (1FL), alkaloids, amino acids, vitamins and some trace elements and Barbary wolfberry oil seed, is a kind of androgen effect of Chinese medicine and can enhance the body immunity, anti-tumor and aging function and increase hematopoietic function. Besides, its active ingredients can regulate the hypothalamus-pituitary-gonadal axis and element metabolism, protect the reproductive system.^[28]^ When it is absorbed into the liver and kidney, it can nourish the liver, tonify the kidney and qi, calm the mind and blood. ^[29]^

Some studies have reported that angelica dahurica extract can block extracellular calcium influx to play a role of relaxing blood vessels. Besides, it also has anticoagulation, antioxidant, free radical clearance and other function.^[29]^

Coix seed contains fatty acids and esters, polysaccharides, brass, triterpenoids, alkaloids, sterols, lactam and other effective ingredients. Many scholars have reported that coix seed can enhance the activity of red blood cell Na^+^ / K^+^ ATP enzyme to maintain the normal morphology of red blood cells, thus improving the blood supply.^[30]^

Moreover, myristerol feruate and vegetable oil sterol feruate in coix seed were able to induce ovulation and stimulate ovarian follicle growth in mice.^[31]^

It is documented that ginger (ginger) may normalize the hormone balance and control the menstrual cycle.

Jujube is rich in polysaccharides, cyclic adenylate, vitamins, inorganic salts, irons and other active ingredients, which can play an important role in the prevention and treatment of anemia.^[32]^ Experimental studies have shown the effects of sedative, antidepressant-like, estrogen and antiprogesterone.

### Menstrual-related TCM syndrome score and efficacy index evaluation

Menstruation-related TCM syndrome score can comprehensively evaluate patients’ menstrual conditions and accompanying symptoms and is a commonly used evaluation index in current TCM clinical research.

Many previous studies have shown that the combined treatment has a good clinical effect in treating oligomenorrhea and hypomenorrhea. Wang et al ^[33]^ proposed that the combined group and the Chinese medicine group were significantly better than the PG group in improving the syndrome of patients with decreased menstruation after surgery, but there was no distinct difference between the COM group and the Chinese medicine group.

In our study, the TCMSS of the three groups gradually decreased over time. However, only in the combined group, the score was significantly lower than that of 1 month. After 3 months of treatment, the TCMSS score of the combined group was significantly lower compared with the PG group. Moreover, the scores of the combined group were reduced greater than PG group, which means the combined therapy may achieve greater effect than mono-therapy.

We further compared the efficacy index of menstruation-related TCM syndrome in each group. The results show after 3 months of treatment, the total response rate of TCM syndrome score in all groups increased significantly compared with 1 month of treatment, indicating that the efficacy of each group was more significant with the extension of treatment time. In addition, the effective rate of the COM group was higher than mono-therapy. Therefore, the combination treatment was superior to the single drug in relieving related symptoms of patients disturbed with oligomenorrhea and hypomenorrhea.

### Menstrual blood loss score

The PBAC score enables a semi-quantitative assessment of menstrual blood loss, with objectivity and flexibility.^[34]^ Liu *et al*, After 3 months of treatment for patients with irregular menstruation caused by polycystic ovarian syndrome, The PBAC scores of the treatment group (given XiaoYao pills combined with cyproterone tablets) and the control group (oral ethinyl estradiol cyproterone tablets) were significantly higher compared with those before treatment, And the most obvious improvement in the patients in the treatment group.

The results of this study showed that the PBAC score of TCM group was significantly higher after 1 month compared with before treatment, and PBAC after 3 months was significantly higher than 1 month after treatment, indicating that the TCM group had an effect quickly and had a significant effect on improving menstrual volume; In the comparison between groups, after 3 months of treatment, the difference and rate of TCM group score change were significantly greater than those of PG group.

There were differences between groups in the difference before and after treatment, which more reflected the group differences in drug efficacy in cases of inconsistent baseline.

Interestingly, the COM group treatment after 1 month score and no obvious difference before treatment, treatment after 3 months score is significantly higher before treatment, and treatment in January and treatment after 3 months score no significant difference, we speculate hormone effect or play its biological active interference, still need to further improve the mechanism of clinical research and basic research.

### General body health status score

Our study shows that SF-36 is commonly used to assess physical and mental health, reflecting quality of life, the higher the SF-36 score, the better the quality of life, the treatment of sparse and reduced menstrual volume is not excellent enough in improving menstrual blood loss, but has a positive impact on patient quality of life. The SF-36 score did not decrease in each group, indicating that the drug had no negative effect on the physical and mental health status of the patients. It is speculated that the improvement of the SF score will occur, but further testing is needed.

### Sex hormone levels and endometrial thickness

The change of sex hormones once again confirms that the TCM group does improve the ovarian reserve function, and PG group has a regulatory effect on prolactin. Prolactin in TCM group did not elevate, so it can be boldly speculated that Chinese medicine can partially offset the side effects caused by progesterone. It is proved that Chinese medicine compound multi-component multi-target, more comprehensive reversal of the disease, is the advantage of TCM group.

In patients with low ovarian function, not only FSH significantly decreased, but also AMH significantly increased, with a significant difference before and after treatment, which is consistent with the report in the literature that TCM can improve ovarian function. The feedback effect of progesterone in the PG group makes the decrease of FSH equally obvious. However, due to the small sample size in the combined group, the changes before and after treatment were not significant. In comparison, the traditional Chinese medicine group can improve the ovarian function very well.

### Metabolomics

In addition, we decided to conduct a post-hoc experiment to further understand the potential mechanism *via* metabolomic profiles. With data pre-processing, the efficient and stable quality of measurement of LC-MS was validated with quality control samples. The clear separation between pre and post treatment within TCM, PG and COM groups were determined with PLS-DA model, suggesting major alternations of patients’ seral metabolism profile have occurred during 3-month treatment. A total of 155, 180 and 176 differential metabolites were identified in TCM group, PG group and COM group, respectively, in which 6 metabolites shared importance in all three regimens, and 30 metabolites involved in both TCM and progesterone monotherapy, and 133 more differential metabolites were identified exclusively for COM treatment, suggesting a diverse targets between TCM and progesterone, even distinct targets in COM treatment.

With KEGG pathway enrichment and topological analyses, GPL metabolism were found significantly altered in all 3 groups, and the improvement of clinical parameters may be closely related to this pathway. GPL are fatty acid diglycerides with a phosphatidyl ester attached to the terminal carbon, dominating the cellular membrane fluidity and permeability, and required for normal function of membrane proteins, receptors and ion channels.^[35]^ The abnormal GPL metabolism has been reported in follicular fluid in patient with polycystic ovary syndrome (PCOS), accounting for the pathogenesis of follicular development disorders.^[36]^ Additionally, in research comparing seral metabolic difference between offsprings of patients with PCOS and healthy controls, the results suggested the major differential metabolites were GPLs.^[37]^ As one of the major symptoms to diagnose PCOS is oligomenorrhea, our results implied that GPL metabolism improvement may closely related to the improvement of clinical characteristics. Furthermore, the enrichment of alternation of GPL metabolism has been reported in circular ribonucleic acid profile of ovarian endometriosis, suggesting endometrial related pathogenesis of GPL metabolism.^[38]^

Tryptophan metabolism alternation was filtered out in KEGG topological analysis in both TCM monotherapy and in combo treatment, suggesting it may be targeted by therapy of TCM. Tryptophan metabolism involved in the kynurenine, 5-hydroxytroptamine, and indole pathways.^[39]^ Many bioactive derivatives generated from tryptophan metabolism exsert regulatory physiological function in inflammation, metabolism, immune response, and neurological function,^[40]^ and the reduced inflammation in adipose tissue has been proved to alleviating obesity and improving insulin sensitivity.^[41]^ In the observatory research comparing metabolomics between PCOS patients and healthy controls, increased tryptophan concentration was observed in all PCOS subgroup, suggesting it may be involved in malfunction in ovulation, presented as oligomenorrhea clinically.^[42]^ It could be further implied that TCM probably could modify the ovulatory function of patients with either oligomenorrhea or PCOS.

### Deficiency and outlook

There were some limitations in our study. Firstly, due to the appearance and properties of different drugs, our study did not carry out a double-blind control design. In addition, there was too few patients with diminished ovarian reserve in this study, which could not reflect the improvement of ovarian reserve well and expand the conclusion to more general population with menstrual disorders. The treatment time of this subject is only 3 months, it only assessing short-term drug efficacy, The long-term efficacy of TCM, PG and COM in patients’ luteal phase endometrial thickness was not examined in this research, and more precisely defined trials are needed for further exploration. Due to patients’ heterogeneity, the external data validation and future trials with a larger sample size were expected to validate the results. Metabolomic methods were first preformed to exploring their common and distinct effects on symptom improvement. The paired statistics analyses were used to perform precise comparison between data before and after 3-month treatment. However, as limits of post-hoc analysis, validation of external data and cell or animal study were required to strengthen the results. Finally, heterogeneous etiology of oligomenorrhea and hypomenorrhea may impair the ability to extrapolate the results.

## Conclusion

In conclusion, the results of the trial showed that the combination of Chinese and Western medicine can significantly reduce the TCMSS scores and have a higher response rate than monotherapy. It also provided preliminary evidence that the TCM is superior to PG in regulating physiology condition in the long run in terms of the PBAC scores, hormone levels and endometrium thickness, especially in patients with Ovarian insufficiency. Therefore, the TCM combined with progesterone capsule is an effective and safe option in treating oligomenorrhea and hypomenorrhea. In metabolomic approach, distinct distributions between pre- and post-treatment were identified among all three groups, and a total of 155, 180 and 176 differential metabolites were identified in TCM group, PG group and COM group, respectively. With KEGG pathway enrichment and topological analyses, GPL metabolism was found significantly altered in all 3 groups, while TCM could potentially improve the clinical symptom *via* tryptophan metabolism. 2 additional pathways were uniquely influenced under combo treatment, namely Riboflavin metabolism and caffeine metabolism, suggesting a synergetic effect of combination TCM with PG in disease management. Metabolomic profile *via* LC-MS was proved to be a promising method to exploring the TCM and progesterone in management of oligomenorrhea and hypomenorrhea, providing insights towards better understand of different underlying mechanisms.
